# Personalising glioblastoma medicine: explant organoid applications, challenges and future perspectives

**DOI:** 10.1186/s40478-025-01928-x

**Published:** 2025-01-11

**Authors:** Niclas Skarne, Rochelle C. J. D’Souza, Helen M. Palethorpe, Kylah A. Bradbrook, Guillermo A. Gomez, Bryan W. Day

**Affiliations:** 1 Sid Faithfull Brain Cancer Laboratory, QIMR Berghofer Medical Research Institute, Brisbane, QLD 4006 Australia; 2 School of Biomedical Sciences and Faculty of Medicine, The University of Queensland, Brisbane, 4072 Australia; 3 Centre for Cancer Biology, University of South Australia and SA Pathology, Adelaide, SA 5000 Australia; 4 School of Biomedical Sciences, Faculty of Health, Queensland University of Technology, Brisbane, 4059 Australia

**Keywords:** Glioblastoma, Brain cancer, Explants, Organoids, Patient-derived, Heterogeneity, Personalised medicine

## Abstract

Glioblastoma (GBM) is a highly aggressive adult brain cancer, characterised by poor prognosis and a dismal five-year survival rate. Despite significant knowledge gains in tumour biology, meaningful advances in patient survival remain elusive. The field of neuro-oncology faces many disease obstacles, one being the paucity of faithful models to advance preclinical research and guide personalised medicine approaches. Recent technological developments have permitted the maintenance, expansion and cryopreservation of GBM explant organoid (GBO) tissue. GBOs represent a translational leap forward and are currently the state-of-the-art in 3D in vitro culture system, retaining brain cancer heterogeneity, and transiently maintaining the immune infiltrate and tumour microenvironment (TME). Here, we provide a review of existing brain cancer organoid technologies, in vivo xenograft approaches, evaluate in-detail the key advantages and limitations of this rapidly emerging technology, and consider solutions to overcome these difficulties. GBOs currently hold significant promise, with the potential to emerge as the key translational tool to synergise and enhance next-generation omics efforts and guide personalised medicine approaches for brain cancer patients into the future.

## Introduction

The past decade has witnessed the rapid evolution of a powerful new translational platform to study human disease: 3D organoid model systems. Advances in our understanding of stem cell biology has resulted in the generation of numerous in vitro organoid cell approaches [1]. By providing stem cells with a set of biochemical and biophysical cues mimicking the in vivo stem cell niche, organoid differentiation protocols allow stem cells to differentiate into respective lineages and self-organise into complex 3D structures resembling the native organ, whilst maintaining self-renewal capability [1]. These approaches were rapidly applied to human disease, in particular cancer, giving rise to patient-derived tumour organoids (PDOs), where tumour cells are dissociated at the single-cell level and subsequently established into organoid structures [2]. In contrast to the PDO technique, patient-derived explants (PDEs) consist of ex vivo culture of intact fragments of resected tumour tissue. 3D culture of fresh animal tissue was pioneered in 1951 by Leighton, with the first human tumour histo-cultures performed by Freeman and Hoffman in 1986 [3, 4]. PDEs are attractive as they maintain tissue architecture, the TME, tumour-specific genetic alterations, transcriptomic profiles and the histopathology of the original patient lesion [5]. An intact TME more accurately reflects real-life biological interactions between normal stroma, the immune system and cancerous tissue, providing a robust translational platform to better model the standards-of-care (SOC) or test novel therapies in a solid tumour setting. In the context of precision oncology, the ease of availability of tumour specimens allows relatively efficient generation of PDEs directly from clinical specimens without complex dissociation protocols and stem cell culture conditions. PDEs are methodologically straightforward to generate, inexpensive to culture, and have been proven to predict patient response when exposed to approved therapies [6, 7]. Recent developments at the federal level in the United States further support the integration of explant systems into clinical management. In December 2022, the U.S. Food and Drug Administration (FDA) waived the requirement that all drugs be tested in rodent and non-rodent animals before human clinical trials [8]. Moreover, in Australia, The Commonwealth Scientific and Industrial Research Organisation (CSIRO), in collaboration with industry, developed a report assessing the potential of emerging non-animal models, defining organoids and explants as critical components to guide treatment decisions for cancer patients [9]. The European Medicines Agency (EMA) has also expanded its actions and regulatory considerations in reducing animal use by supporting the development of alternative technologies including organoids and organ-on-chip models during drug development [10]. These important advances in legislation will support the ongoing use and development of explant and organoid systems in preclinical oncology, aligning this emerging technology with the clinical trials sector into the future. In this review, we summarise the current applicability and standing of preclinical models in brain cancer, focusing on the emerging importance of patient-derived glioblastoma (GBM) explant organoid models (GBOs). We analyse the key advantages and limitations of this rapidly developing technology and discuss the potential of GBOs to parallel the patient journey and revolutionise personalised medicine approaches in this intractable disease setting.

## Single-cell derived GBM model systems

GBM is the most common and aggressive malignant adult primary brain cancer and is associated with a median survival of approximately 15 months [11]. Since there has hardly been any change in GBM treatment modalities for decades, new strategic approaches for the identification and development of novel drug targets are urgently required. GBMs are highly heterogeneous tumours, comprising drug resistant infiltrative tumour cell populations that exist in a complex immune and vascular TME [12–14]. A recent single-cell RNA sequencing (scRNA-seq) study, conducted by Neftel and colleagues, established multiple transcriptional cell-states underlying GBM heterogeneity. These cell-states, termed neural progenitor-like 1 (NPC1), neural progenitor-like 2 (NPC2), oligodendrocyte progenitor-like (OPC), astrocyte-like (AC), mesenchymal-like 1 (MES1) and mesenchymal-like 2 (MES2), contain distinct genetic drivers and were shown to be highly plastic [14]. In addition, it is well-established that GBM cells interact with various non-malignant cell types within the TME, contributing to treatment resistance and immune evasion [15–17]. This biological complexity underscores the need for model systems that accurately reflect the true disease state. The overwhelming majority of prior and current drug development efforts rely heavily on 2D cell autonomous in vitro systems combined with lead-candidate validation in patient derived xenograft (PDX) models.

### 2D GBM models

In GBM, 2D systems typically comprise of commercially available cell lines (e.g. U-251 MG and U-87 MG) and primary patient GBM derived cell lines [18–20]. The main advantages of 2D cell lines are ease of culture, maintenance and manipulation, lower costs and the power of enabling reproducible high throughput screening [19, 20]. In addition, patient derived primary models more closely mirror the heterogeneity of the parental tumour when compared to commercial cell lines, when cultivated over low passage numbers [21]. These 2D systems, however, are usually monocultures and allow for the study of only one cell type, lack the TME, or niches, which in vivo may be required by cancer-initiating glioma stem cells (GSCs). In addition, GSC culture conditions, including the use of exogenous EGF/bFGF and/or serum to propagate cells over serial passages, select the fastest, most dominant clones or AC/MES-like cell-states [22, 23]. The culture supplementation of EGF/bFGF is also known to result in the loss of EGFR variant III (EGFRvIII) and EGFR amplification, two prevalent oncogenic GBM-drivers. These in vitro complexities should be taken into consideration when studying the molecular events of GBM malignancy and drug screening [24].

### Patient-derived xenograft (PDX) models

These models consist of dissociated primary tumour cells that are directly injected, typically into immuno-compromised mice. The major advantage of PDX xenografts is the ability to phenocopy intra-tumoural heterogeneity and preserve tumour architecture [25]; features not present in 2D culture systems. For example, Kerstetter-Folge et al.. demonstrated that orthotopic PDX models maintain remarkable histological similarity to the patient tumour throughout the first three in vivo intracranial passages. They showed that cell morphology and growth characteristics of GBM tumours such as pseudo-palisading necrosis, microvascular abnormalities and infiltrative growth patterns were retained in PDX tumours [26]. Similarly, Kitange et al.. demonstrated that PDX models also maintain *MGMT* promoter methylation, an important predictor of temozolomide (TMZ) response [27]. Joo et al.. showed that copy number variations and mutations identified in patient tumours were precisely replicated in their matched PDX counterpart [28]. These studies have highlighted the usefulness of PDX models nevertheless, they do present key limitations. Tumour engraftment rates can be highly variable ranging from 10 to 100%, with long latency, high cost, administrative ethics burden, and issues pertaining to animal welfare [29]. While PDXs can broadly recapitulate the polygenomic architecture of human tumors, they do not fully account for heterogeneity in the TME. The presence and extent of pro and anti-tumor environments, and tumor associated macrophages (TAMs), in PDX models remain uncertain. Most PDX models also use immuno-deficient mice, lacking a complete functional immune system. It is well established that the immune system plays crucial roles in brain cancer treatment response, especially to immunotherapy [30]. This limitation can be overcome by conducting experiments in humanised ‘immune-competent’ mouse models, but these approaches are costly and can give rise to issues of immune-compatibility (species-dependent host vs. graft rejection) [31, 32]. An attractive alternate immune-competent animal model is the genetically engineered mouse model (GEMM) that employs immunocompetent mice where one or more genes involved in malignant transformation are deleted, mutated, or overexpressed [33]. Even though this model offers a more realistic immune TME, GEMMs cannot reproduce the complexity or heterogeneity of cancers.

### 3D cerebral organoids

**Fig. 1:**
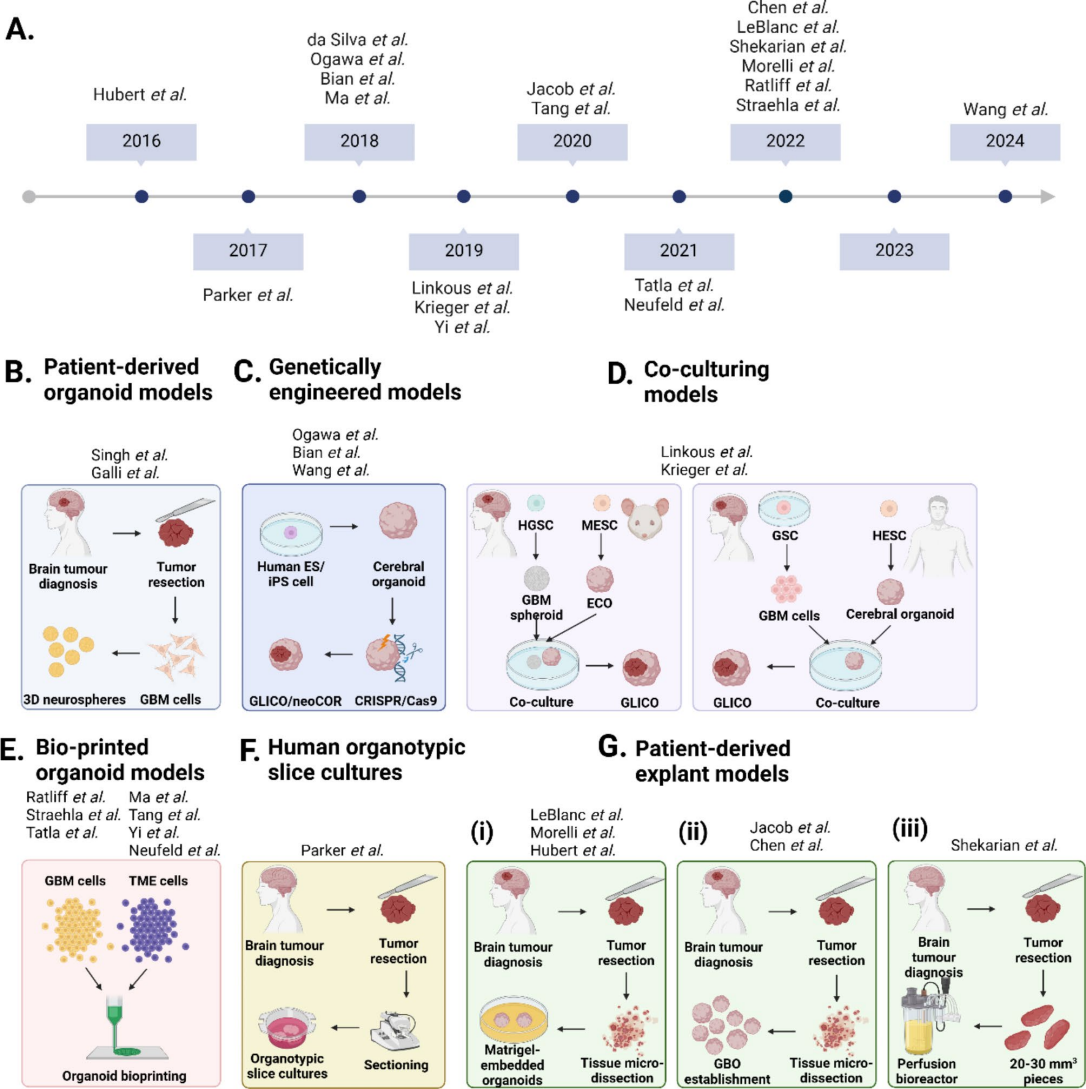
The History of 3D Brain Tumour Model Development. A timeline illustrating the evolution of various 3D preclinical models in GBM (**A**). Models include: PDOs (**B**), genetically engineered models (**C**), co-culturing models (**D**), bio-printed organoids (**E**), organotypic slice cultures (**F**) and PDEs (**G**). Abbreviations: embryonic stem cell (ES cell), cerebral organoid glioma (GLICO), neoplastic cerebral organoids (neoCOR), human glioma stem cell (HGSC), mouse embryonic stem cell (MESC), glioma stem cell (GSC), human embryonic stem cell (HESC). Created with BioRender.com

The last decade has seen significant effort and progress made in the development of 3D organoid systems to model GBM (Fig. 1A). Galli and colleagues were the first to adopt neurosphere culture conditions to generate GSC-rich ‘glioma-spheres’, a PDO model which maintained cell polarisation and a degree of spatial organisation (Fig. 1B) [34, 35]. These glioma-spheres are established from pure populations of dissociated GSCs and thus lack normal TME-associated cell interactions [2]. The introduction of induced pluripotent stem cells (iPSCs) and their ability to generate organotypic models with multicellular composition and structural organisation has attracted great attention in the field of neuro-oncology. Cerebral organoids are pluripotent stem cell-derived 3D systems that display the structure and organisation similar to that of a foetal brain [36–39]. To be able to model GBM in this setting, researchers developed neoplastic cerebral organoids (neoCORs), wherein glioma-genesis was induced by genetic manipulation of known GBM-drivers [40–42] (Fig. 1C). These organoid approaches have, thus far, been limited to known ‘classical-truncal’ canonical GBM-driver mutations, therefore incompletely replicating tumour heterogeneity. Linkous et al.. and Krieger et al.. attempted to overcome this issue with the development of the cerebral organoid glioma (GLICO) model, where GSCs are co-cultured with iPSC-derived human cerebral organoids (Fig. 1D). This impactful model demonstrated GSC proliferation, invasion, and integration into the iPSC-derived organoids [43, 44]. The main limitation of all iPSC-derived organoid models is the lack of a functioning TME, the long duration for establishment and the high cost of maintenance [45].

### Bio-printed 3D cultures

A rapidly developing and novel field is the bio-printing of complex tissues (Fig. 1E) [46–52]. Tang and colleagues have integrated multiple cell types to establish a “tetra-culture” [50]. Neufeld and colleagues aimed to tackle the problem of models lacking tumour-stroma interactions by generating bio-printed organoids consisting of GBM cells, astrocytes, microglia and perfusable blood vessels consisting of brain pericytes and endothelial cells [52]. Key limitations of this model system include the lack of normal brain tissue, the requirement of specialised equipment and expertise, and the homogeneity of printing substrates. These factors still impede widespread adoption of these technology platforms.

## GBM explant model systems

### Slice cultures

Culturing resected tissue provides a relatively simpler alternative to iPSC-derived or bio-printed organoids, naturally preserving native cell architecture and intrinsic complex tumour-stroma biological interactions. Early approaches centred on the development of organotypic brain slice cultures [53] and human GBM organotypic slice cultures [54] (Fig. 1F). These models preserve critical features of the host tissue such as glial-neuronal interactions and neuronal connectivity and provide an authentic extracellular matrix (ECM). In addition, human organotypic slice cultures closely recapitulate the human TME. These models generally have a short lifespan of only a few days, and their development is both technically challenging and time-consuming [55].

### Explant cultures

These approaches involve the ex vivo culture of intact fragments, generated by cutting the resected tumour tissue into pieces of approximately one to several millimetres in diameter, without mechanical or enzymatic dissociation under a dissection microscope. The pieces are placed in culture dishes or flasks, with defined media and/or basement membrane matrices, allowing the intact tissue to grow, adhere and migrate. This temporarily preserves the original cytoarchitecture, native cell-cell interactions and TME of patient tumours. This approach overcomes the short lifespan of slice cultures [56]. Glioma explant models evolved from research conducted in the early 90’s by Bjerkvik and colleagues, who established ‘organotypic tumour spheroids’ by seeding small tumour pieces in agar-coated flasks [57]. From this early experiment, numerous methodologies have been developed (summarised in Table 1). Hubert et al. reported a method to culture minced GBM tissue, embedded in Matrigel as 3D ‘organoids’ [23, 58, 59]. These organoids are composed of diverse stem and non-stem cell tumour populations, which were more representative of the parental lesion than spheroids derived from patient-derived cell lines (Fig. 1Gi). Jacob et al. was the first to establish the seminal explant GBO model that is the main focus of this review [60–62]. This PDE approach consists of culturing intact pieces of GBM tumour tissue on an orbital shaker to facilitate GBO formation and enhance nutrient and oxygen diffusion (Fig. 1Gii). GBOs typically take two weeks to establish in culture, accompanied with the acquisition of a spherical morphology and can be subsequently cryopreserved. The GBO approach is being widely adopted as the current state-of-the-art by many laboratories worldwide, due to key advantages. Firstly, GBOs retain features of high-grade gliomas as well as histological features of the parental tumour. These include (i) cellular morphology and nuclear atypia, (ii) CD31-positive vasculature, a known stem cell niche in brain cancer [61], (iii) cellular diversity, (iv) gene expression signatures and transcriptional patterns comparable to the parental tumour, and (v) the frequency and distribution of the Neftel cell-states [60, 61]. This was also shown by LeBlanc et al., with GBO cell-state proportions remaining consistent following expansion [23]. A second advantage of the GBO system is the presence of the TME; albeit transiently, with non-neoplastic cells such as macrophages/microglia, T-cells, stromal cells, and myelinating oligodendrocytes surviving for up to two weeks in culture [60]. Thirdly, GBOs are also able to reproduce GBM properties in vivo when transplanted into adult immune-deficient mouse brains, showing extensive infiltration into surrounding, normal brain tissue [61]. Finally, GBOs can be faithfully cryopreserved, following 2–4 weeks of culture, enabling bio-banking and efficient recovery while retaining expression patterns to the corresponding parental tumour upon recovery [61]. While the model defined by Jacob et al. has paved the pathway for PDEs, GBOs are size limited due to the diffusion limit of oxygen and nutrients [61]. Shekarian and colleagues described an extension of the GBO model where larger pieces of GBM tumour tissue were cultured as GBM explants in 3D perfusion bioreactors [63] (Fig. 1Giii). This system provides a continuous flow of media throughout the tissue, allowing larger tissue pieces (20–30 mm^3^) to be propagated that maintained immune cells and the TME for up to three weeks.

**Table 1:** 3D brain cancer model systems

	Year	Author	Material	Methodology	Tumour model	Generation time	TME
Traditional 3D culture	2016	Hubert et al. [59]	patient-derived GSCs	Tumour pieces/cells embedded in matrigel	GBM	> 2 months	No
	2019	Frisira et al. [65]	Patient-derived GSCs	Tumour cells embedded in matrigel, followed by orbital shaker	MB	4 weeks	No
	2020	Jacob et al. [60]	GBM tumour pieces	3D culture on orbital shaker, no ECM/EGF/bEGF	GBM	1–2 weeks	Yes
	2020	Loong et al. [66]	Patient-derived GSCs	Tumour cells embedded in matrigel	GBM	10 days	No
	2022	Chen et al. [67]	GBM tumour pieces	3D culture on orbital shaker	GBM	1–2 weeks	Yes
	2022	LeBlanc et al. [23]	GBM tumour pieces	Tumour pieces embedded in matrigel	GBM	17–55 days	Yes
	2022	Shekarian et al. [64]	GBM tumour pieces	Perfusion bioreactor	GBM	1–2 weeks	Yes
	2022	Morelli et al. [58]	GBM tumour pieces	Tumour pieces embedded in matrigel	GBM	3 days	Yes
	2022	Abdullah et al. [68]	LGG tumour pieces	3D culture on an orbital shaker, no ECM/EGF/bEGF	LGG	1–2 weeks	Yes
	2022	Sundar et al. [69]	Patient-derived GSCs	Tumour cells embedded in matrigel	Paediatric HGG	1 week	No
	2023	Lago et al. [70]	Tumour pieces	3D culture on an orbital shaker, EGF + FGF-2	EPN MB LGG	1–2 weeks	Yes
	2023	Verduin et al. [71]	GBM tumour pieces	Tumour cells embedded in matrigel	GBM	> 2 months	No

## Advantages of GBO explant model systems

### Applicability for in vitro drug efficacy screens

There is now a growing focus on translational studies testing novel therapeutics in GBOs. Technically, this is feasible and scalable, providing a viable model that maintains the characteristics and composition of the parental tumour [61, 70]. GBO propagation enables access to live cultures on-demand to examine drug effects on proliferation, apoptosis, cell-cell interactions, the influence of immune cells in the TME (type, density, function) and biomarker analysis. In addition, unlike patient-derived xenografts (PDXs), which are time-consuming and costly, GBOs can be established in a few weeks and be employed almost immediately to generate viable translational data, with the potential to impact patient care in real time. Numerous positive studies have already provided strong support for the use of GBOs in translational research [60, 71–77] (summarised in Table 2). Recent notable examples include studies undertaken to assess the efficacy of chimeric antigen receptor T (CAR-T) cell therapies using GBO-CAR-T co-culture assays. GBOs also maintain the expression of specific glioma-associated antigens such as EGFRvIII, which is known to be lost in 2D culture, especially cultures supplemented with EGF [60]. A proof-of-concept study conducted by Jacob et al. demonstrated the ability of GBOs to be utilised as an ex vivo testing platform for immunotherapy [60, 75]. CAR-T cells were shown to invade GBOs, and expansion of EGFRvIII-specific T cells was observed within organoids with high EGFRvIII levels. Specific CAR-T cell mediated killing was further demonstrated by cleaved-caspase 3 staining and increased presence of granulated T cells in proximity of EGFRvIII-positive cells. Another study has recently reported effective GBO targeting using a CAR-T directed towards the tumour-associated receptor tyrosine kinase EphA3 [78]. EphA3 is known to be associated with GSCs in the perivascular space and MES subtype GBM [79, 80]. Using GBO-CAR-T co-culture assays, Martins and colleagues demonstrated that EphA3 CAR-T cells effectively infiltrated, disaggregated and induced apoptosis in EphA3-positive GBOs [78]. Numerous other studies have examined the activity of small-molecule drugs in GBOs, evaluating on-target activity and anti-tumour responses [74, 76, 81]. Darrigues et al. applied the GBO model to evaluate the effectiveness of small-molecule inhibitors that were previously shown to be effective at inhibiting tumour cell invasion in 2D cultures. This proof-of-concept study demonstrated that GBOs present an attractive option for assessing invasion inhibition in larger patient cohorts and paves the way for similar future studies [73]. GBOs have also been used to investigate strategies to overcome resistance to TMZ. By co-culturing glioma-associated fibroblasts (GAFs) with GBOs, Zuo et al. were able to show that GAFs promoted TMZ chemo-resistance through C-C motif chemokine ligand 2 (CCL2)-mediated signal-regulated kinase 1/2 (ERK1/2) activation. By treating GBOs with inhibitors targeting CCL2 or mitogen-activated protein kinase 1/2 (MEK1/2), chemo-resistant GBM cells could be re-sensitised to TMZ [77]. A similar approach was used by Cui et al., who demonstrated that oxyphyllanene 1 sensitises TMZ-resistant cells in both GBM cell lines and GBOs [82].

**Table 2:** GBO drug efficacy studies

YEAR	AUTHOR	MODEL	STUDY
2020	Jacob et al. [60]	GBO	EGFRvIII-targeting CAR-T cell co-culture with GBOs.
2021	Lenin et al. [73]	GBO	Screening drugs on GBOs pre-treated with the SOC for personalised treatment for recurrent glioblastoma.
2021	Thokala et al. [74]	GBO	Comparing the efficacy of EGFRvIII-targeting CAR-T cell constructs using GBO model.
2021	Darrigues et al. [75]	GBO	Screening a library of 22 compounds that inhibit tumour invasion into surrounding tissue.
2021	Bouché et al. [83]	GBO	Validating anti-tumour activity of quisinostat treatment.
2022	Wei et al. [76]	GBO	Investigating the therapeutic efficacy of Gamitrinib.
2022	Song et al. [77]	GBO	Demonstrating the potential of combining the IAP antagonist Birinapant with CAR-T cells to overcome tumour antigen heterogeneity in GBOs.
2022	Cui et al. [84]	GBO	Investigating whether Oxyphyllanene-B treatment is effective against TMZ-resistant cells.
2022	Chen et al. [62]	PDO	Testing the sensitivity of PDOs to different therapeutics in parallel to the SOC.
2023	Li et al. [78]	MBO	Evaluating the efficacy of CT-179 anti-tumour effects in MBOs.
2024	Zuo et al. [79]	GBO	Investigating whether targeting the CCL2-CCR2 axis or MEK1/2-ERK1/2 pathway effectively improves the therapeutic efficacy of TMZ.
2024	Martins et al. [80]	GBO	Investigating the efficacy of EphA3-targeting CAR-T cells using GBO model.

### Applicability for personalised medicine

Despite advances in the development of novel anti-cancer therapies, drug combinations and accurate patient selection, limited progress has been made in aggressive cancers such as GBM. A key limitation to this is the ‘one-size-fits-all’ paradigm, and “all-comer” clinical trials where drug agents result in less than 50% response in patients [83]. The concept of precision medicine provides a potential avenue, where patient-specific abnormalities are identified using next generation omics approaches, and drug agents targeting these aberrations, if available, are administered to the patient. However, interim analysis of ongoing precision oncology trials, such as NCI-MATCH [84] or NCI-IMPACT [85], has revealed that these targeted approaches often result in disappointing clinical outcomes with lower-than-expected progression-free survival. These observations can be explained by the role of non-genetic mechanisms driving cancer hallmarks such as growth and drug resistance [5, 84]. For example, the TME and its spatiotemporal altering complexity and importance in drug resistance and chemo-sensitivity is not considered in these static, genomics-based therapeutic approaches [86, 87]. In contrast, functional precision oncology enables drug efficacy testing using in vitro preclinical models. An increasing number of studies in other cancer types have been harnessing the advantages of PDE culture [88–93]. This is of particular importance for preclinical testing of immunotherapies, as these agents require an intact, human-specific microenvironment to be fully functional [5]. This is being increasingly recognised by the neuro-oncology field, with many laboratories making effective use of the GBO platform in their discovery research (Table 2). By leveraging explants, valuable insights can be gained into individualised drug responses, resulting in more effective and tailored treatment regimens for cancer patients. An ex vivo model that accurately reflects patient responses could therefore facilitate the identification of the druggable ‘Achilles heel’ of an individual cancer. The aggressive nature of GBM, the short life expectancy and limited therapeutic window, are all challenges to personalised medicine strategies. The GBO model possesses major advantages due to the short timeframe for generation and assay duration. This allows for candidate drug identification, early in the treatment journey, well before the patient succumbs to tumour relapse. GBOs have been found to recapitulate patient-specific responses to treatment when subjected to similar post-operative treatments including radiation and TMZ therapy. GBOs, which were most resistant to therapy, corresponded to patients with below median survival, and GBOs that demonstrated treatment sensitivity corresponded to patients with extended survival [61]. GBOs established from patients with GBM, a metastatic brain tumour, or low-grade glioma were also found to be resistant to SOC when treated for 48 h. In this study, utilising GBOs, the authors identified the most effective treatment options to achieve favourable outcomes in these patients with tumour relapse [62]. Although the above studies have shown the potential of GBOs in predicting patient response, large-scale, proof-of-principle studies are yet to be conducted.

### Applicability for omics analysis

To further expand the translational potential of GBOs, the amalgamation with pan-omics technologies will be beneficial. In modern cancer research, pan-omics data is an essential component providing a more complete picture of underlying cancer biology. Utilising omics for 3D in vitro model studies enables tumour model validation, heterogeneity studies and a mechanistic understanding of tumour pathophysiology, as well as treatment response [94].

**Fig. 2:**
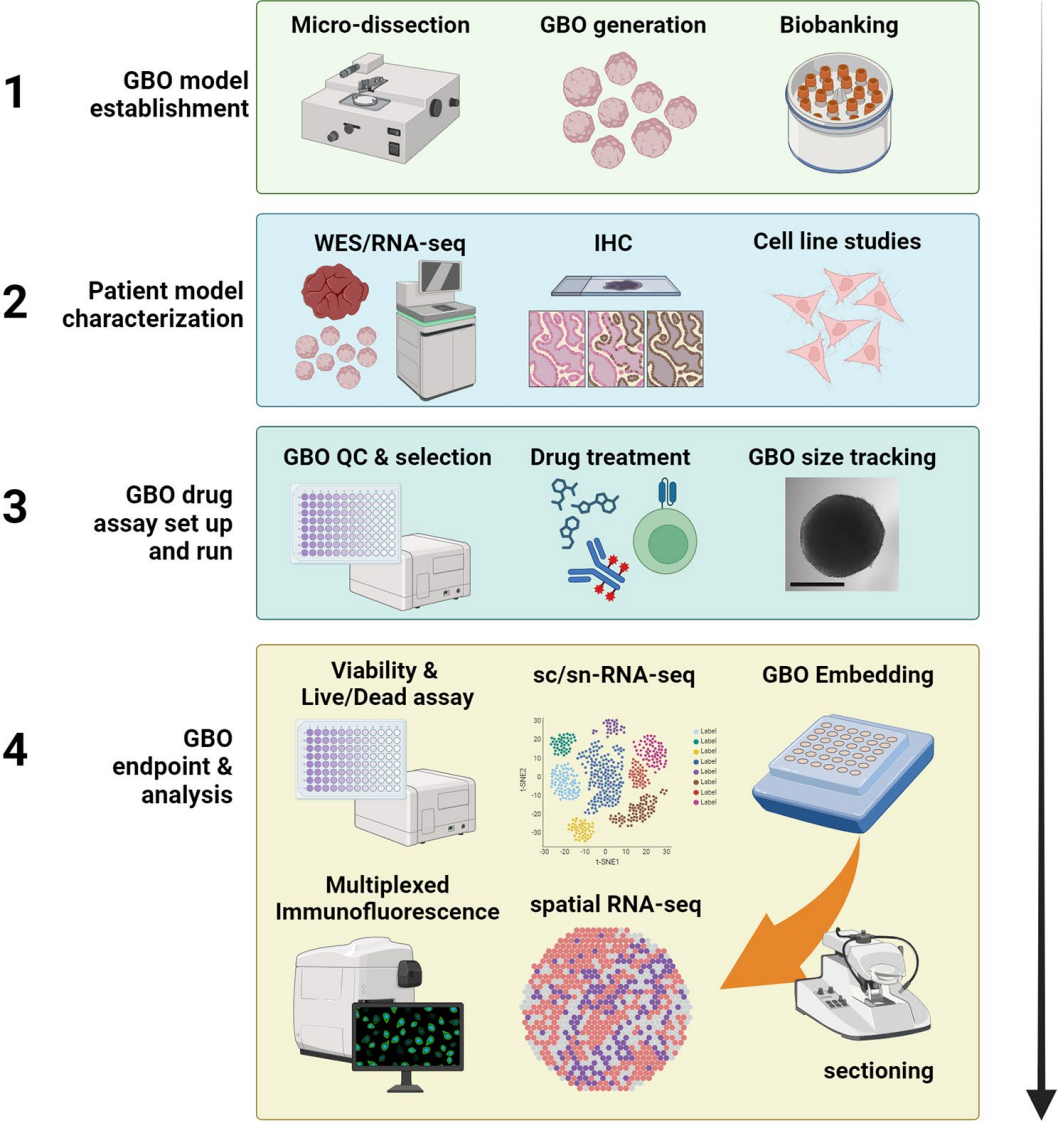
GBO drug assay approaches. Step 1: GBO model establishment - involves microdissection and processing of the tumour tissue, GBO generation and biobanking. Step 2: Patient model characterisation - constitutes of genomic and transcriptomic characterisation of tumour. Drug target and/or biomarker expression can be examined by IHC staining on tumour tissue. Studies in the matching, patient-derived cell line may also be performed to select candidate drugs to take forward for testing in the GBOs. Step 3: GBO drug assay preparation – includes GBO QC, which can be performed to select viable GBOs prior to drug treatment commencement. During treatment exposure, GBO size can be tracked as a measure of treatment efficacy via bright field microscopy. Step 4: GBO endpoint analysis – this constitutes of live-cell readouts prior to GBO dissociation for scRNA-seq or embedding and downstream analysis by techniques such as immunofluorescence microscopy and spatial RNA-seq. Abbreviations: Whole exome sequencing (WES), Immunohistochemistry (IHC), Quality control (QC), single nuclei (sn), single cell (sc). Created with BioRender.com

scRNA-seq can be applied to GBOs to assess transcriptional states of non-neoplastic cells, tumour heterogeneity and the TME. However, dissociation of cells can result in altered abundances or in the loss of specific cell types. Alternatively, single-nucleus RNA sequencing (snRNA-seq) can be employed, which implements harsher conditions to release free nuclei, overcoming many dissociation-related artefacts [95]. In the context of the brain mounting evidence suggests that many genes act locally at synapses, known as the dendritic transcriptome. These important cell interactions could be lost using single cell approaches [96, 97] highlighting the need for studying tumours in situ. Spatial transcriptomics is a good choice for GBO analysis, as the spatial relationship of tumour cells and the TME is retained [98]. Cell multiplexing allows cells of each individual GBO to be labelled with a molecular tag and analysed in batches. Miles and colleagues effectively employed spatial profiling, of individual PDEs, to co-register drug responses with tumour pathology, heterogeneity and the TME using multi-fluorescence combined with digital image analysis [99]. GBOs can be readily paraffin-embedded or cryopreserved in OCT matrix allowing for multiple time points to be assessed at the completion of the experiment. Recent innovations in spatial proteo-transcriptomics is revolutionising our ability to understand tumour biology and mechanisms dictating therapy resistance and response. Spatial transcriptomics using instruments such as the 10x Genomics Visium and ultra-multiplex spatial proteomics using the CODEX/Phenocycler enable unprecedented biological insights into drug-induced changes in gene expression relative to the TME, cellular organisation or potentially targetable ligand-receptor interactions between neighbouring cells [98, 100, 101]. Furthermore, spatial omics data could also be integrated with histology images to build deep learning models predictive of drug response [102]. The integration of PDE culture with spatial omics technologies and machine learning approaches will be increasingly adopted in future studies (Fig. 2, Step 4).

GBOs are typically generated from numerous different geographical regions within the tumour and thus exhibit significant regional heterogeneity. Different regions are shaped by distinct genetic and epigenetic drivers, regional transcriptional programs, micro-environmental ques etc [103, 104]. These factors result in spatial heterogeneity of gene expression, somatic mutations, copy-number aberrations, and chromosomal rearrangements across the spectrum of the lesion [105]. Therefore, a drug or therapy examined in a particular assay would likely result in a range of responses within GBOs derived from different regions of the same specimen. GBOs could therefore be treated as ‘mini-tumours’ and profiled individually. Nevertheless, the inclusion of sufficient biological GBO replicates is required to adequately address heterogeneity and determine on-target drug specificity. Shekarian and colleagues assessed the effects of immunotherapy on the immune TME by implementing highly multiplexed microscopy (CODEX, *co*-detection by in*dex*ing) for spatially resolved cell identification. In this study, they discovered significant differences in expression levels of functional molecules in immune cell types between responding and non-responding PDEs. In addition, significant differences in composition and therapy response between explants derived from either the tumour core compared to the periphery, were also observed [63]. Correlating expression to response also requires assay termination, at the optimal time, to capture both the efficacy of the drug with sufficient viability remaining to perform target expression or cell death analysis. From our laboratory experience, we have observed that assessing drug response at later time points can result in lower drug target expression due to the elimination of target-positive cells [76].

## Limitations of GBO explant model systems

### Establishment

GBO generation relies heavily on obtaining sufficient viable fresh tumour tissue in a timely fashion, posing a logistical and ethical hurdle. Jacob and colleagues reported variable results in GBO maintenance and expansion across specimens and considered both tissue viability and tumour composition to be major factors contributing to these observations [61]. Tissue quality is affected by numerous practical factors, including the time elapsed post-collection, storage conditions and intracranial tumour location, which affects the extent of tumour resection. Tumour composition, including the abundance of necrotic tissue, percentage of tumour versus normal and degree of vascularisation of the resected tumour piece, are also critical. Growth characteristics and the genetic aberrations of the parental tumour also play a role in successful GBO establishment. Jacob and colleagues reported IDH1-WT tumours with an aggressive growth phenotype to have a significantly higher success rate (96.4%) when compared to IDH1-mutant tumours (66.7%) [61]. This study also reported a reduced success rate for recurrent tumours (75%). This could, in-part, be due to the increased immune infiltrate in recurrent tumours, or resected tissue comprising of regions of diffusely infiltrated normal brain [106]. The reduced GBO success rate experienced with lower-grade gliomas (LGGs), IDH1-mutant and recurrent tumours suggests that further optimisation and refinement of the GBO protocol will prove useful.

### Protocol modifications

There have been attempts to apply the GBO protocol for the establishment of PDEs derived from LGGs. However, this has had limited success and requires additional modifications to the original protocol described by Jacob et al. [68]. PDEs from other cancer types such as medulloblastoma (MB) have been established using the original protocol [76], however, LGGs and other paediatric brain cancers such as diffuse midline gliomas (DMGs) have required modification. Abdullah and colleagues reported an adaptation to the GBO protocol which enables efficient establishment of PDEs from LGGs [66]. By modifying the protocol to incorporate GBO establishment under conditions closer to physiological oxygenation they were able to demonstrate LGG organoid generation with a success rate of 87% for WHO Grade 1–3 tumours. Lago and colleagues established patient-derived organoids from ependymomas, MBs and low-grade glial tumours. Importantly, they compared their protocol to published culture conditions adopted by Jacob et al. and Abdullah et al. Here they found supplementation of EGF and bFGF to be critical for PDE establishment. They argued that for GBOs, often established from GBM specimens harbouring EGFRvIII, that these factors are unnecessary. However, for other types of brain cancer, these factors appeared vital for PDE establishment. Concerning LGG tumours, this study also found that paediatric LGG PDEs did not propagate well with the protocol established by Abdullah et al. This is consistent with the notion that adult LGGs and paediatric LGGs are clinically and molecularly distinct diseases, with the latter usually being less aggressive [107]. Patient-derived meningioma organoids have now also been successfully established using the initial protocol from Jacob and colleagues, albeit with minor modifications. These meningioma organoids showed a stronger resemblance to parental tumours when compared to previous meningioma models [108].

### Applicability for modelling recurrence

Multiple studies into tumour evolution have shown that recurrent tumours vary widely from the primary counterpart [109–112]. An argument against the use of GBOs, as a personalised medicine platform, is that the primary GBO may not effectively model relapse. A potential solution to overcome this limitation is to apply the selective pressure of radiation and/or chemotherapy, to mimic post-operative treatments given to patients. This strategy has already been employed by Lenin and colleagues [71]. Here, they aimed to identify an effective treatment for recurrent GBM by testing the efficacy of candidate compounds on GBOs that had been subjected to prior in vitro SOC treatment for GBM. This study reported the agent costunolide, a TERT inhibitor, to be effective in reducing viability of both primary GBOs and GBOs pre-treated with radio-chemotherapy. However, the short GBO treatment timeline of approximately one week (50 µM TMZ and 10 Gy) used in this study may not accurately reflect the longer SOC timeframe currently given to GBM patients (6 weeks of TMZ and 60 Gy of radiotherapy). This shorter window, to apply SOC treatments in GBOs, is a key limitation that the broader field is actively attempting to resolve.

### Loss of the immune TME

GBOs display decreasing numbers of the cellular components of the TME such as macrophage/microglia populations and reduced vasculature, as well as reduced expression of immune-related markers [23, 61]. GBOs are also at risk of clonal drift over prolonged culture, causing a loss of molecular and cellular diversity. Jacob and colleagues observed the gradual loss of the macrophage/microglia marker IBA1 and T cell marker CD3 over time [60]. This was argued to be due to defined culture conditions, which were optimised to specifically preserve tumour cell viability and proliferation. The loss of the immune TME can be especially detrimental when examining the efficacy of immunotherapies such as CAR-T cells. One practical solution is to assay the GBO as early as possible after establishment, the strategy of ‘fresh-is-always-best’ in our hands and many others, is the most efficient and expedient way to generate reliable translational data using GBOs [78].

The second solution, to the loss of immune cells, involves the modifying of culture conditions. This approach could be useful when attempting to run GBO assays for longer time periods of several weeks. For example, microglia viability is dependent on interleukin-34 (IL-34), colony-stimulating factor 1 receptor (CSF1R) and transforming growth factor β (TGF-β) [113, 114]. By supplementing 100 ng/mL IL-34, CSF (5 ng/mL), and TGF-β (50 ng/mL) into the medium, Hong et al. were able to support microglia-containing cerebral organoids [114]. It is therefore likely that the addition of these factors would similarly help the propagation of microglia in GBOs. However, modifying culture conditions only maintains viability of the specific immune population but may not drive proliferation.

An attractive alternative to combat the shortcoming of declining immune TME, is to exogenously reconstitute cell types. There are three main sources from which immune cells can be sourced: differentiated blood and bone marrow cells, iPSC derived cells or direct isolation from the tissue of interest. Miller et al. investigated the immunomodulatory activity programs of glioma-associated myeloid cells by co-culturing GBOs with human myeloid CD11b and CD45-positive cells, isolated from tumour or donor patient peripheral blood mononuclear cells (PBMCs). Here GBOs showed extensive infiltration with myeloid cells, which upregulated canonical microglia markers, demonstrating that bone-marrow derived monocytes acquired features of tissue resident microglia [115]. Abud et al. demonstrated that microglia-like cells can be differentiated from iPSCs, which when transplanted into cerebral organoids, matured, ramified, and responded to injury. More detailed insights into the use of such immune co-cultures have been described elsewhere [116–118]. While these approaches can repopulate immune cells in 3D models, a significant amount of research is still required to be able to precisely tailor culture conditions and design methodologies to reproducibly maintain phenotype and longevity. Studies co-culturing immune cells isolated from iPSCs or the tumour have yet to be demonstrated in GBOs. Despite these obstacles, it is likely that future advancements in organoid technology will expand the boundaries of GBO lifetime and enhance suitability for longer assays requiring the presence of an intact TME. Advancing GBO-immune co-culture models also holds great potential in preclinical immunotherapy development and could be applied for predicting clinical response to different immunotherapies.

### Limited throughput capability

In comparison to other single-cell based in vitro approaches, GBO establishment can be time-consuming and involve significant manual processing [61]. This is mainly due to dissection of the whole tumour into one millimetre sized pieces under a microscope. We have recently trialled benchtop tissue processing with positive results, the McIlwain Tissue Chopper (TC752), prepares uniformly sized and viable tissue cubes in a matter of minutes (Fig. 2, Step 1) [119]. This approach is reproducible and can be standardised at multiple sites to align with multi-site clinical trials efforts. Another key issue is limited starting tumour material that effects the number of GBOs generated. Most GBO studies, described earlier, use small numbers of GBO replicates (*n* = 3), raising questions of appropriate coverage with respect to tumour heterogeneity [60, 78]. A potential solution is to develop a parallel matched 2D primary cell line for initial high throughput bulk analysis, followed by validation of key findings in the matched GBO (Fig. 2, Step 2) [71]. An alternate, but less attractive approach, involves increasing GBO number through several rounds of expansion, leading to increased loss of immune cells and the TME. Importantly, in the case of drug assays, target antigen expression or pathway activation should be correlated to individual GBO response. This strategy overcomes small replicate numbers and provides key readouts which can be correlated to specific molecular pathways, TME targeting and cell death response. By utilising more sophisticated analytical methods, the complex ecosystem of explants and compositional change can be dissected, garnering more data per individual GBO (Fig. 2, Step 4). For example, Palethorpe et al. described an optimised PDE drug screening platform involving single-cell transcriptomics coupled with proteomics to examine intra-tumoural heterogeneity and cellular composition changes of individual GBOs following drug treatment [119]. Even though a low GBO replicate number was used, deep insights into the mechanism of action of preclinical compounds were obtained.

### Assay challenges

Most explant studies to-date make use of destructive end-point assays, such as basic IHC/immunofluorescence (IF), and global viability or gene expression analyses such as quantitative PCR (qPCR). A simple non-destructive readout involves tracking GBO size over the course of the experiment using bright field image capture (Fig. 2, Step 3). This enables a quick and easy method of determining the efficacy of a given drug or therapy over time [61]. A limitation is that live and dead tissue cannot be differentiated using this approach. Apoptotic tissue often remains attached to the GBO, which can skew final results. In addition to size assessment, viability assays can be performed to quantitate therapy response. The CellTiter-Glo^®^ reagent is well-suited for 3D micro tissue cultures. This is a powerful tool used to predict health, growth/size and energy status of 3D tissue and has been utilised in a range of organoid models [120–122]. Non-toxic viability assays such as PrestoBlue™ (Thermo Fisher Scientific) have been widely used for 2D cell culture assays, and suitability for 3D cultures has also been demonstrated [88, 123, 124]. In addition, these live-cell approaches can be applied as a quality control surrogate of viability before commencement of further investigational studies (Fig. 2, Step 3). In an alternate approach, we have shown the effective use of NucBlue™ Live ReadyProbes™ (Thermo Fisher Scientific) and propidium iodide for live/dead analysis of GBOs in response to drug exposure [119]. The non-destructive NucBlue™ Live ReadyProbes™ approach additionally allowed us to perform post-hoc cell death analysis using the CellEvent™ Caspase-3/7 ReadyProbe™ reagent (Thermo Fisher Scientific). Taken together, live-cell approaches are viable and if employed effectively, can combine multiple GBO proliferation and cell death readouts throughout the course of the experiment.

## Conclusion

**Fig. 3:**
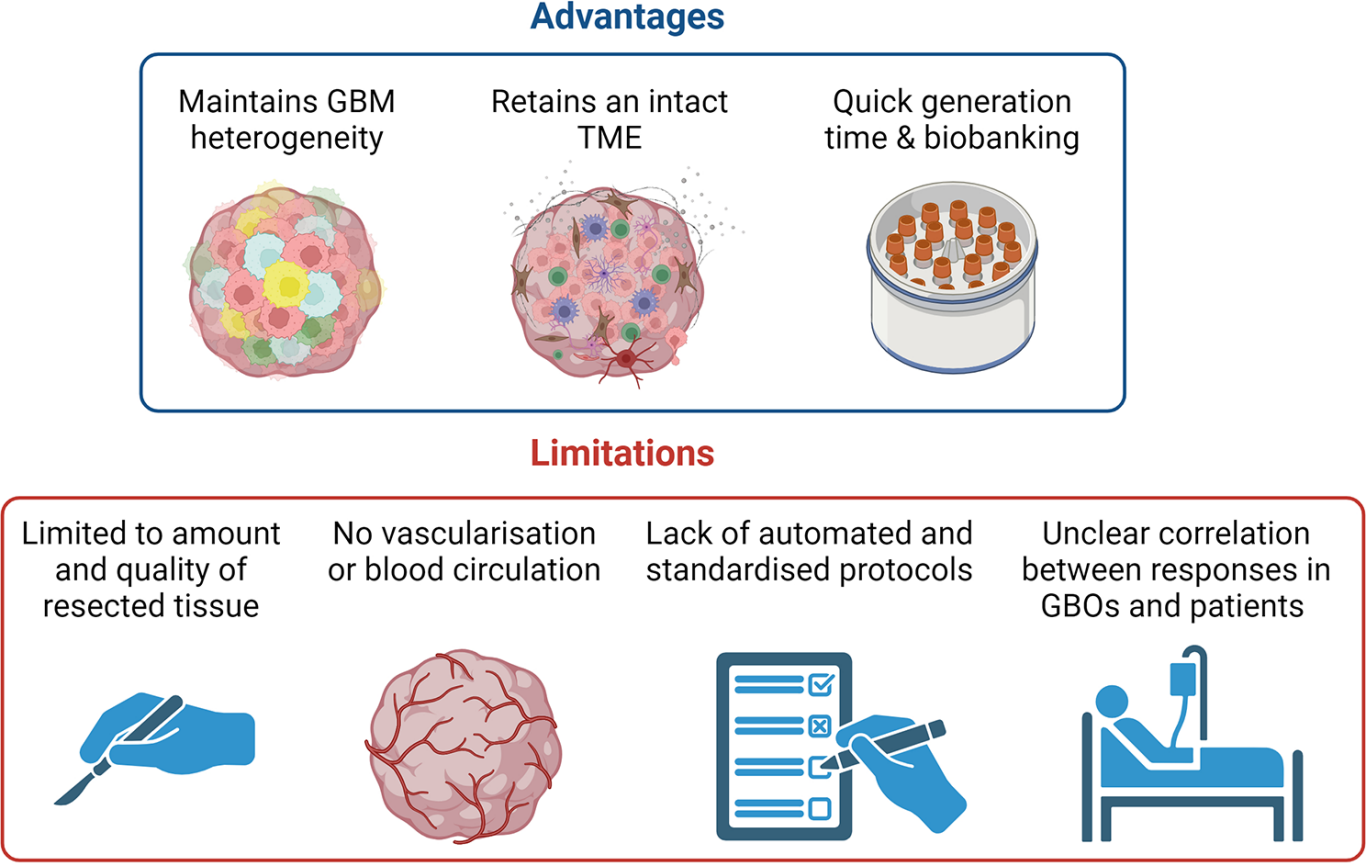
The current pros and cons of GBOs. Advantages include the retention of GBM heterogeneity, an intact TME, the quick generation time and the ability to biobank GBOs, and the apparent correlation between responses in GBOs and patients. Limitations include insufficient amount and quality of resected tissue, the lack of vascularisation and blood circulation, the absence of standardised and automated workflows for GBO assays and the inability to replicate clinical treatments due to the limited lifespan of GBOs. Abbreviations: TME: tumour microenvironment. Created with BioRender.com

GBOs are evolving as a translationally relevant, patient-proximal ex vivo platform to examine the sensitivity of drugs or SOC therapies. GBOs present several key advantages over present technologies, retaining tumour architecture, heterogeneity and the TME of the parental tumour. An emerging body of evidence now supports GBOs as a viable surrogate of the parental tumour, enabling studies examining cell-cell interactions, the TME during tumour progression, selective therapeutic pressure, as well as the effect of the TME on therapeutic response and resistance. This is especially relevant when examining the efficacy of immunotherapies. Integrating GBOs into an efficient workflow, enabling multiple readouts in parallel with emerging omics technologies will be critical to overcome current limitations of this exciting and emerging technology (summarised in Fig. 3). Future efforts should focus on large-scale, proof-of-principle clinical studies to definitively establish and correlate patient responses to the paired companion GBO. This final hurdle will define clinical applicability, standardise accreditation processes and ultimately facilitate the future applicability of GBOs to guide personalised medicine approaches in intractable diseases such as brain cancer. In summary, 3D organoid technologies have long-held huge burgeoning potential. GBOs finally appear to be on the precipice to deliver on this important translational promise.

## Data Availability

No datasets were generated or analysed during the current study.

## Abbreviations

3D: 3-dimensional
AC: Astrocyte-like
BBB: Blood brain barrier
CAR-T cell: Chimeric antigen receptor T-cell
CCL2: C-C motif chemokine ligand 2
CSF1R: colony-stimulating factor 1 receptor
DMG: Diffuse midline gliomas
ECM: Extracellular matrix
ERK1/2: Signal-regulated kinase 1/2
ES: Embryonic stem cell
GAF: glioma-associated fibroblasts
GBM: Glioblastoma
GBOs: Glioblastoma organoids
GLICO: Cerebral organoid glioma
GSC: Glioma stem cell
HESC: Human embryonic stem cell
HGSC: Human glioma stem cell
IDH: Isocitrate dehydrogenase
IHC: Immunohistochemistry
IL-34: interleukin-34
iPSC: Induced pluripotent stem cell
LGG: Low grade glioma
MB: Medulloblastoma
MEK1/2: Mitogen-activated protein kinase 1/2
MESC: Mouse embryonic stem cell
MES1: Mesenchymal-like 1
MES2: Mesenchymal-like 2
mIF: multiplexed immunofluorescence
neoCOR: Neoplastic cerebral organoids
NPC1: Neural progenitor-like 1
NPC2: Neural progenitor-like 2
OPC: Oligondendrocyte progenitor-like
PDO: Patient-derived organoid
PDE: Patient-derived explant
PDX: Patient-derived xenograft
QC: Quality control
scRNA-seq: single-cell RNA sequencing
sc: single-cell
sn: single-nuclei
SOC: Standard of care
TGF-β: transforming growth factor β
TME: Tumour microenvironment
WES: Whole exome sequencing
